# Public Perceptions on the Use of the Physical Activity Readiness Questionnaire

**DOI:** 10.3390/healthcare12171686

**Published:** 2024-08-23

**Authors:** Anantharaman Venkataraman, Ian Zhirui Hong, Lisa Cuiying Ho, Tess Lin Teo, Stefanie Hwee-Chee Ang

**Affiliations:** 1 Department of Emergency Medicine, Singapore General Hospital, Emergency Medicine Academic Clinical Program, Duke-NUS Academic Medical Centre, Outram Road, Singapore 169608, Singapore; ian.hong.zr@gmail.com (I.Z.H.); tess.teo.lin@singhealth.com.sg (T.L.T.); 2 Department of Orthopaedic Surgery, Sengkang General Hospital, 110 Sengkang East Way, Singapore 544886, Singapore; ho.lisa.ho@gmail.com; 3 Sport Cares, Sport Singapore, 3 Stadium Drive, Singapore 397630, Singapore; Stefanie_ANG@sport.gov.sg

**Keywords:** self-administered pre-participation survey, physical activity, co-morbidities, feedback

## Abstract

Self-administered pre-participation screening for physical activity (PA) requires an instrument that should be easily used and identify individuals at high risk. The Physical Activity Readiness Questionnaire (PAR-Q+) has been used for many years. Its ease of use and ability to identify those not fit to undergo PA has not been assessed. This study was to determine the rates of the PAR-Q+ in identifying adults who may not be fit for moderate or intense PA and obtain feedback on the use of this tool. A randomized, cross-sectional study involving a wide spectrum of members of the public was carried out. Participants were asked to provide their bio-characteristics, complete the PARQ+, and provide feedback on the questionnaire. With 1019 participants, about 33.1% of the participants using the PARQ+ would have required further medical evaluation. Except for those patients with respiratory illness, there was no difference in levels of PA in those who answered yes or no to the seven PARQ+ questions. Only 4 of the 7 main PAR-Q+ questions were perceived by the public as easily understood. Difficulties were encountered with 21 of the 45 follow-up questions, especially amongst those with co-morbidities. The wordiness of the questions and the large number of technical terms were also sources of concern. Suggestions were provided by participants on areas where improvements may be made to the wording of the questions. The study suggests that the PAR-Q+ probably over-identified those who require further medical evaluation. In addition, the wordiness of the questions and frequent use of medical jargon made the PARQ+ challenging to understand and use. The suggestions provide opportunities to review areas for possible improvements.

## 1. Introduction

While exercise reduces health risks in the medium and long term, the risk of sudden death is transiently increased during or immediately after physical activity (PA). There have been many instances worldwide where sports enthusiasts performing either moderate or intense PA have sustained cardiac arrest or other significant physical injuries during or immediately after the activity [1,2]. Because of the need to identify those likely to sustain such injury and take preventive measures, a Physical Activity Readiness Questionnaire (PAR-Q) for use by all persons wishing to increase their level of PA was developed in Canada in 1970 and endorsed by Fitness Canada and the American College of Sports Medicine. This tool has been used in many countries [3]. The document has undergone multiple revisions since then, the latest version being the 2021 PAR-Q+ [4].

In 2007, a sports safety committee was set up to address the occurrence of adverse events during sporting activities recommended the adoption of the PAR-Q (then a one-page document) or its appropriately amended version as the principal pre-participation PA readiness tool for the country [5]. Over the years, in spite of this recommendation, injuries during moderate and intense sports, as a result of pre-existing conditions, continued to occur. The frequency of use of the PAR-Q or its variations as a pre-participation tool by those engaging in sporting activities was also low. In Canada, too, a new tool, the Get Active Questionnaire (GAQ), has been proposed to replace the PAR-Q+ [6].

The current version of the PARQ+ is a four-page document to be completed by the exercise participant. The participant would be required to answer seven questions on page 1 of the PAR-Q+. A no answer to all of the questions’ clears the participant for unrestricted PA following the general guidelines for healthy asymptomatic populations [7,8,9]. A participant answering yes to 1 or more of the questions would need to complete pages 2 and 3, which contain follow-up questions on specific chronic medical conditions, to either clear the respondent or refer to an online ePARmed-X+ to be completed by a medical practitioner. Advice is also provided on developing a safe and effective PA plan, including recommendations appropriate for lower-risk individuals with established chronic medical conditions. At the end of the ePARmed-X+ process by the medical practitioner, the participant might be cleared for unrestricted or partially restricted PA participation.

There were many concerns about the continued use of the PARQ+. One was that it may result in too many medical referrals that will inundate the healthcare system. Having many persons undergo medical evaluation before engaging in physical activity would be a disincentive to exercise [10]. In addition, if members of the public have difficulties understanding the main and follow-up questions in the PARQ+, its use may not be useful for them [11,12]. Feedback from the public could potentially be helpful in considering the use of alternate questionnaires. With additional concern at the increasing length and complexity of self-administering pre-participation screening programs in the local and other populations [13] and the continuing occurrence of adverse outcomes from moderate to intense PA in spite of these pre-participation questionnaires, the authors, a sub-group of the Sport Safety Workgroup, undertook a review of pre-participation screening questionnaires with a view to reconsidering the use of such tools by sports participants in the country.

The objectives of this study were to determine the ability of the PARQ+ to identify adults requiring further medical evaluation prior to moderate or high-intensity PA, assess their difficulty in using this tool, and obtain community feedback on this.

## 2. Materials and Methods

This was a randomized, analytical cross-sectional study on the use of three PA pre-participation questionnaires. The PAR-Q+ arm was included as one of the three owing to it having been the first major tool recommended by Sport Singapore for at least fourteen years and generally used in many countries around the world. The study was conducted as a public survey.

Prior to doing the survey, participants were provided with basic information on the potential need for PA pre-participation screening and the need for a locally relevant questionnaire. Formal consent to participate in the study was then obtained. Upon recruitment, potential participants were randomized to one of the three available such questionnaires, one of which was the PAR-Q+. On being randomized to the PARQ+ arm, participants were asked to complete three sections. The first was on personal bio-characteristics, including age, gender, height, weight, ethnic group, occupation, social habits such as cigarette smoking, and frequency and duration of their PA habit. The second section was the completion of the PAR-Q+ by the participant. The third was a questionnaire on the participant’s ease or otherwise in understanding each of the seven questions and the follow-up questions that followed in the event of a “yes” answer, and also on the use of technical terms in and the length of the questionnaire. The feedback was on a 5-point Likert scale. The participants were also asked whether the questionnaire needed additional items of information and their preferred frequency for completion of the PAR-Q+.

The study was powered to show a 10% difference in mean values with a standard deviation of 25% using α = 0.50 and *p* = 0.05. The minimum required for a comparison of each group was 293. However, with the need to consider at least three subgroups of respondents within each arm of the study (by age-group, occupational status, and presence of co-morbidities), we multiplied this number by 3 giving a total of 879. With the expectation of at least 10% incomplete survey responses, the minimum number required per study arm was increased to 1000.

The study was conducted via an online survey platform, SogoSurvey^TM^ by Sogolytics^TM^ 25.0 (Herndon, VA, USA).

The survey was open to any interested member of the public over the age of 21 years. Publicity about the study was carried out through various national sports organizations, community grassroot organizations such as the Peoples Association, governmental agencies such as the Health Promotion Board, professional groups like the Academy of Medicine, and institutions of higher learning. Participation was voluntary, and participants had to agree to participate in order to proceed.

The study was approved by the Institution Review Board of the Singapore Sport Institute.

### Data Analysis

All data collected were anonymized and transferred automatically to a Microsoft Excel Version 2407 database. The analysis was carried out using IBM SPSS Statistics 21.

For comparative analysis, patients were divided into three age groups (viz., young adults aged 21 to 39.9 years, middle-aged adults aged 40 to 59.9 years, and elder adults aged 60 years and above). The participant’s body mass index (BMI) was calculated, and the participants were grouped by BMI using the Asian BMI scale suggested by the World Health Organization [14]. Participants were also grouped into 10 main occupational codes based on the International Labour Organisation classification of occupational codes [15]. They were then further re-grouped into three groups: Group 1 was for those in managerial and professional occupations; Group 2 was for those with administrative and clerical duties; and Group 3 was for the retired, unemployed, housewives, manual workers, and military personnel. Based on their declared level of current PA, they were also grouped into those with low PA (<150 min per week), moderate PA (150–299 min per week), high PA (300–449 min per week), and very high PA (≥450 min per week).

The authors considered the influence of satisficing in feedback data entry in this online survey. We considered five types of satisficing behaviors amongst the survey respondents, viz., primary bias, acquiescence bias, early termination, non-response, and straight-lining. The satisficing analysis was performed as per the methods suggested by Barge, Vriesema, and Gehlbach [16,17]. The analysis suggested an estimate of 62% occurrence of various forms of satisficing, especially of the straight-line type. We initially removed the apparently satisficed data from the third section’s feedback analysis, calculated the means of the Likert scores, and used these to obtain four groups of levels of difficulty. This resulted in the use of a Likert mean score of ≥4.51 to suggest ease of answering the question, 4.01–4.50 as slight difficulty, 3.51–4.00 as moderate difficulty, and ≤3.50 as significant difficulty.

Descriptive statistics were used for nominal and ordinal data. Non-parametric comparisons were made using independent samples (Kruskal–Wallis test and Mann–Whitney U test), as appropriate. Chi-square tests were used for parametric data comparisons and ANOVA for comparisons between groups.

## 3. Results

A total of 1020 persons were randomized to the PAR-Q+ study arm. Of these, 1 was omitted from further analyses because of extensively incomplete data. The analysis was based on the remaining 1019 responses.

### 3.1. Participant Characteristics (Table 1)

These are given in Table 1. The ethnic breakdown of the participants was not significantly different from that of the general Singapore population. Amongst all study participants, 57.8%. reported exercising for at least 150 min per week.

The BMI was higher in the 21–39.9 and 40–59.9 age groups compared to those over 60 years (24.8 vs. 23.8, *p* = 0.012). About 43.4% had at least one illness. Participants’ age was directly related to the presence of co-morbidities (*p* = 0.000). Those in the older age groups spent more time on PA (292 min vs. 207 min for those <60 years old, *p* = 0.000). Those with co-morbidities spent 210 min per week on PA versus 233 min for those without known co-morbidities (*p* = 0.075). There was a decrease in PA duration with increasing numbers of co-morbidities (*p* = 0.014). Those with BMI < 18.5 and >33.0 had lower levels of PA than those with values between 18.5 and 32.9 (153 vs. 219 min, *p* = 0.000). For occupational groups, those in Group 3 exercised more than those in Groups 1 and 2 (265 min vs. 214 and 208 min, *p* = 0.001).

**Table 1:** Characteristics of study participants.

Participant Characteristic	Data	Based on June 2022 Singapore Population Data (Where Available)
Gender (%)		
Male	621 (60.9%)	48.8%
Female	398 (39.1%)	51.2%
Age (mean ± sd ^1^)	46.9 ± 12.1 years	47.8 years
Age Distribution		
21–39.9 years	298 (29.2%)	35.6%
40–59.9 years	539 (52.9%)	38.0%
60–80.0 years	182 (17.9%)	26.4%
Cigarette Smoker (%)		NA ^3^
Yes	65 (6.4%)	
No	954 (93.6%)	
Body Mass Index (BMI)		NA ^3^
Very low (<18.5)	36 (3.5%)	
Low (18.5–22.9)	344 (33.8%)	
Moderate (23.0–27.4)	430 (42.2%)	
High (27.5–32.9)	170 (16.7%)	
Very high (>33.0)	39 (3.8%)	
Ethnic Groups		
Chinese	847 (83.1%)	75.3%
Malay	65 (6.4%)	12.8%
Indian	83 (8.1%)	8.7%
Others	24 (2.4%)	3.2%
Occupational Groups (by ILO ^2^)		NA ^3^
Occupation Groups 1–2 (managers and professional groups)	366 (35.9%)	
Occupation Groups 3–5 (clerical, administrative and executives)	420 (41.2%)	
Occupation Groups, 7–9, 0 (manual, retired, homebased, military)	233 (22.9%)	
Duration of Physical Activity per week (minutes)		NA ^3^
0–149 min	430 (42.2%)	
150–299 min	312 (30.6%)	
300–449 min	158 (15.5%)	
≥450 min	119 (11.7%)	
Physical Activity duration (minutes) by Age Group		*p* = 0.000 ^4^
21–39.9 years	204 ± 216	
40–59.9 years	209 ± 200	
≥60 years	292 ± 216	
Physical Activity duration (minutes) by BMI		*p* = 0.000 ^4^
≤18.5	164 ± 161	
18.5–22.9	226 ± 202	
23.0–27.4	220 ± 211	
27.5–32.9	211 ± 239	
≥33	141 ± 210	
Physical Activity duration (minutes) by Occupational Group		*p* = 0.001 ^4^
Occupational Groups 1–2	214 ± 187	
Occupational Groups 3–5	208 ± 219	
Occupational Groups 7–9, 0	265 ± 232	
Age Group of Respondents with co-morbidities		NA ^3^
No co-morbidities = 577 (56.6%)	45.2 ± 11.6 years	
With co-morbidities = 442 (43.4%)	50.7 ± 12.0 years	
Proportion of co-morbidities by age group:		
Age Group 21–39.9 years	93/298 (31.2%)	
Age Group 40–59.9 years	236/539 (43.8%)	
Age Group ≥ 60 years	110/182 (60.4%)	
By number of co-morbidities		NA ^3^
1 co-morbidity	321/1019 (31.5%)	
2 co-morbidities	101/1019 (9.9%)	
3 or more co-morbidities	20/1019 (2.0%)	

Legend: ^1^ sd = standard deviation; ^2^ ILO = International Labour Organisation; ^3^ NA = not available; ^4^ *p* values determined by use of independent samples Kruskal–Wallis test.

### 3.2. Participants’ Responses to the Seven PAR-Q+ Questions and Their Follow-Up Questions (Table 2)

#### 3.2.1. PAR-Q+ Question 1 (Has Your Doctor Ever Said That You Have a Heart Condition or High Blood Pressure?)

For this first question, 20.9% said “yes”. Increasing age correlated positively with a higher incidence of hypertension or heart problems (*p* = 0.00). Of these, 37.6% had difficulty controlling their condition with medications. There was no significant difference (*p* = 0.069) in the duration of PA for those with or without hypertension or heart disease.

**Table 2:** Participants’ response to PAR-Q+.

1.	Par-Q+ Question 1	
	Numbers answering Yes to Question 1	213 (20.9%)
	b. Numbers with Heart or Cardiovascular Condition Difficulty controlling condition with medications or physician-prescribed therapies Irregular heart beat that requires medical management Chronic Heart failure Diagnosed coronary artery disease and no regular physical activity last 2 months	66 24 12 3 2
	c. Numbers with high blood pressure Difficulty controlling condition with medications or physician therapies Resting blood pressure ≥ 160/90 mm Hg	146 56 28
	d. Numbers answering No to all follow-up questions	22
2.	PAR-Q+ Question 2	
	Numbers answering Yes to Question 2	23 (2.3%)
	b. Numbers with a Respiratory Disease Difficulty controlling condition with medications or physician-prescribed therapies Ever had low blood oxygen at rest or during physical activity and doctor said requires supplemental oxygen If asthmatic, any symptoms of chest tightness, wheezing, laboured breathing, consistent cough > 2 days/week or required rescue medication > twice last week High blood pressure in blood vessels of lungs	5 1 0 0 0
	c. Numbers answering No to all follow-up questions	18
3.	PAR-Q+ Question 3	
	Numbers answering Yes to Question 3	33 (3.2%)
	b. Numbers who have had a stroke Difficulty controlling condition with medications or physician-prescribed therapies Impairment in walking/mobility Stroke last six months	4 2 0 1
	c. Numbers experienced blackout, fainted, lost consciousness with head injury or diagnosed consussion within last 12 months	4
	d. If having low resting blood pressure significant enough to cause dizziness, light headedness, and/or fainting	9
	e. Any epilepsy or neurological condition	2
	f. Numbers answering No to all follow-up questions	21
4.	PAR-Q+ Question 4	
	Numbers answering Yes to Question 4	129 (12.7%)
	b. Numbers with kidney problems	4
	c. Any medical condition not listed above	102
	d. Having 2 or more medical conditions	41
	e. Numbers answering No to all follow-up questions	0
5.	PAR-Q+ Question 5	
	Numbers answering Yes to Question 5	203 (19.9%)
	b. Any metabolic conditions (including Type 1 Diabetes, Type 2 Diabetes, Pre-diabetes) Difficulty in controlling blood sugar levels Often suffering from signs and symptoms of hypoglycemia Any signs/symptoms of diabetes complications Other metabolic conditions	52 17 3 5 4
	c. Planning to engage in unusually high or vigorous intensity exercises in near future	74
	d. Any cancer of any kind Does Cancer diagnosis include lung, multiple myeloma, head and/or neck Currently receiving cancer therapy	6 2 1
	e. Any medical condition not listed above	87
	f. Currently living with 2 more more medical conditions?	111
	g. Numbers answering No to all follow-up questions	1
6	PAR-Q+ Question 6	
	Numbers answering Yes to Question 6	172 (16.9%)
	b. Any arthritis, osteoporosis or back problems Difficulty controlling condition with medications or physician-prescribed therapies Any joint problems causing pain, recent fracture or fracture from osteoporosis/cancer, diaplaced vertebra and/or spondylolysis Any steroid injections or taken steroid tablets for maore than 3 months	67 6 25 1
	c. Any spinal cord injury Difficulty controlling condition with medications or physician-prescribed therapies Any sudden bouts of Autonomic Dysreflexia	5 0 0
	d. Numbers answering No to all follow-up questions	105
7.	PAR-Q+ Question 7	
	Numbers answering Yes to Question 7	10 (1.0%)
	b. Any Mental Health Problems of Learning Difficulties? Difficulty controlling condition with medications or physician-prescribed therapies Any Down Syndrome and back problems affecting nerves or muscles?	1 0 0
	c. Numbers answering No to all follow-up Questions	9

Irregular heart beat requiring medical management was reported by 18.2% of participants in this group. They showed no difference in the duration of PA versus those without an irregular heart rhythm (*p* = 0.980). Only 3 patients had chronic heart failure, and 2 patients with coronary artery disease did not do any physical exercise in the previous two months.

Hypertension was noted by 146 (68.6%) participants from this group, of whom 38.4% had difficulty controlling it with medications or other therapies, and 28 (19.2%) had a blood pressure ≥ 160/90 mm Hg with or without medication. Amongst these two latter groups, there was no significant difference in the duration of PA per week.

#### 3.2.2. PAR-Q+ Question 2 (Do You Feel Pain in the Chest at Rest, during Your Daily Activities of Living or When You Do Physical Activity?)

For the second question, 23 (2.3%) reported chest pain either at rest or during PA. The chest pain group spent an average of 99.1 ± 113.3 min per week on PA versus 226.4 ± 214 min for those without chest pains (*p* = 0.000). All chest pain patients reported having high blood pressure.

Only 5 of the chest pain participants reported having a respiratory illness such as chronic obstructive pulmonary disease, bronchial asthma, or pulmonary hypertension. These spent less time on PA than those without respiratory disease (median 20 vs. 90 min, *p* = 0.227). Another 4 had bronchial asthma, with significant limitation to their weekly PA duration. Amongst all patients with respiratory disease, the duration of PA averaged 178 min vs. 233 min for those without co-morbidities (*p* = 0.029).

#### 3.2.3. PAR-Q+ Question 3 (Do You Lose Balance Because of Dizziness or Have You Lost Consciousness in the Last 12 Months?)

For the third question, 33 (3.2%) said “yes”. There was no significant difference in age or PA level between those with or without dizziness or loss of consciousness.

Only 4 of these 33 patients reported a stroke or transient ischemic attack previously; 4 had experienced a blackout, fainting spell, or loss of consciousness as a result of head injury within the previous 12 months, and 9 (27.3%) exhibited low blood pressure significant enough to cause dizziness, light headedness or fainting. These did not significantly affect the duration of PA they had per week.

#### 3.2.4. PAR-Q+ Question 4 (Have You Ever Been Diagnosed with Another Chronic Medical Condition Other than Heart Disease or High Blood Pressure?)

For the fourth question, 129 (12.7%) said “yes”. Those with chronic medical conditions were older (54.8 vs. 46.5 years, *p* = 0.000). Hyperlipidemia and diabetes mellitus were the most common diseases reported. Only 4 reported kidney problems without these significantly affecting their exercise duration. The medical conditions, per se, did not generally limit their weekly PA duration, unless they had multiple co-morbidities.

#### 3.2.5. PAR-Q+ Question 5 (Are You Taking Prescribed Medications for a Chronic Medical Condition?)

For the fifth question, 203 (19.9%) said “yes”. Of these, 52 had diabetes mellitus, 6 had a cancer and 87 had some non-listed medical condition. Those with at least two medical conditions exercised less (192 vs. 227 min, *p* = 0.033). About 32.7% of diabetics had problems with sugar control, though this did not specifically, statistically, decrease their exercise duration (*p* = 0.075). Those with diabetic complications (9.6% with either hypoglycemia or target organ damage) tended to exercise for a longer duration (median 240 vs. 120 min), though this difference was not statistically significant (*p* = 0.629). The 4 participants with other metabolic conditions, (pregnancy-related diabetes, chronic kidney disease, and liver problems) had no significant differences in their PA duration (*p* = 0.159).

For all 74 who were planning to engage in unusually high or vigorous intensity exercise in the near future, their current level of PA did not differ from those without such plans (*p* = 0.949).

Only 6 respondents reported cancers. Their exercise duration did not differ from those without cancers (*p* = 0.944).

#### 3.2.6. PAR-Q+ Question 6 (Do You Currently Have, or Have Had within the Past 12 Months, a Bone, Joint, or Soft Tissue (Muscle, Ligament, or Tendon) Problem That Could Be Made Worse by Becoming More Physically Active?)

For this sixth question, 172 (16.9%) said “yes”. Of these, 18.6% had upper limb problems, 54.7% had lower limb problems, 12.2% had problems with the trunk, and 14.5% had non-specific aches and pains. There was no significant difference in the PA levels of these four groups of participants (*p* = 0.751). Up to 39.0% of these persons had back and joint pains, which had no impact on PA duration (*p* = 0.477). There was no significant age difference between those with or without joint problems.

Only 6 (0.5%) reported difficulty controlling their condition with medications or other physician-prescribed therapies. While those with difficulty generally exercised less (94 vs. 186 min), this did not reach statistical significance (*p* = 0.326). Up to 37.3% of those with joint problems reported pain, a recent fracture caused by osteoporosis or cancer, displaced vertebra, or spondylolysis. Amongst such respondents, 1.5% required steroid injections. Only 2.9% reported a history of spinal cord injury, which did not significantly impact their duration of PA (*p* = 0.616). None exhibited autonomic dysreflexia.

#### 3.2.7. PAR-Q+ Question 7 (Has Your Doctor Ever Said That You Should Only Do Medically Supervised Physical Activity?)

For the seventh question, 1.0% reported “yes”. Even then, there was no significant difference in PA levels (*p* = 0.916). Only 10.0% of these (or 0.1% of all respondents) reported having any mental health problems or learning difficulties. None reported Down’s syndrome or back problems.

#### 3.2.8. Participants Requiring Further Referral

Overall, 442 (43.4%) of the participants said “yes” to at least one question. Of these, 105 said “no” to all the follow-up questions. Based on the criteria for referral, at least 337 (33.1%) of this cohort would have required referral to a doctor or qualified exercise professional for the use of the ePARmed-X+ or further evaluation. When distributed by age, this would be 15.4% of those aged 21–39.9 years, 34.5% of those aged 40–59.9 years, and 57.2% of those aged 60 years and above. Increasing BMI was associated with increased rates of referral for medical evaluation (*p* = 0.000). Those who were in Occupational Groups 7–9, viz., the retired, unemployed, and housewives, had a higher referral rate for further evaluation than those in Groups 1 to 5 (*p* = 0.000). If those who had difficulty controlling their symptoms were considered (though this was not the basis for the PARQ+ referral for medical evaluation), 111 (10.9%) would likely have required a referral to a medical practitioner for further evaluation.

### 3.3. Participants Feedback on the PAR-Q+

#### 3.3.1. Level of Ease in Understanding the PAR-Q+ Questions

The level of ease in understanding the main questions or the follow-up questions, where applicable, and based on the revised Likert scores, after addressing satisficing by survey participants, is shown in Table 3. Of the main 7 questions, 4 were noted to be easily understood and 3 with slight difficulty. The follow-up question that consistently demonstrated moderate difficulty for 5 of the 7 main questions was “Do you have difficulty controlling your condition with medication or other physician-prescribed therapies?”. The question that generated the overall lowest mean Likert score was “Do you have a resting blood pressure ≥ 160/90 mm Hg with or without medication?”. Out of the 45 follow-up questions, 15 were easy to understand, 14 were encountered with slight difficulty, 3 with moderate difficulty, and 4 with significant difficulty. For 9 follow-up questions, there were no responses to derive any measure of difficulty.

Generally, ease of understanding the questions was lower amongst those with co-morbidities, though this was rarely statistically significant, except for Question 1. There was no significant difference in levels of understanding any of the seven PARQ+ questions by age group, BMI, or occupational group.

The rate of comments obtained from the survey (Table 4) varied from 11.0% for Question 2 to 24.1% for Question 1. For the first six questions, participants who answered “yes” to the main question raised more queries, with this trend being statistically significant for Questions 1, 3, 4, and 6. For Question 7, none of the 10 participants who answered “yes” raised any comments. The comments and suggestions received from the participants are summarized in this table.

The comments and suggestions received from the participants are summarized in Table 5.

#### 3.3.2. Questionnaire Length

Overall, 40.0% of the participants said that the questionnaire length was just right, 50.1% felt it was too long, and 9.9% that it was short. Though more of those with co-morbidities felt that the questionnaire was too long, the difference was not statistically significant (*p* = 0.164). However, there were many recommendations from the participants to shorten the length of the PAR-Q+ questions.

#### 3.3.3. Use of Technical Terms

Up to 46.0% of participants felt that the amount of technical terms used was just right; 46.9% felt that there were too many technical terms used; and the remaining 7.1% felt more terms could be added. Most of those who mentioned excessive use of medical jargon commented on the lack of understanding of these terms by themselves and, by extension, the general members of the community. There was no significant difference in the feedback from occupational groups. However, those with co-morbidities preferred less technical terms (*p* = 0.002).

#### 3.3.4. Suggested Frequency for Completion of the PAR-Q+ Form

The proportion of participants who preferred the PAR-Q+ to be used by exercise participants not more than once annually was 61.9%, while 25.7% preferred it to be carried out once every six months and the remaining 12.4% as frequently as needed. There was no significant difference in the preference by number of co-morbidities (*p* = 0.883) or by age group (*p* = 0.384).

## *4.* Discussion

This study was the first that feedback from reasonably large numbers of members of the public had been obtained on the use of the PARQ+. The study was performed to determine the likelihood of exercise participants needing referral for medical evaluation by using the PAR-Q+ if they were to increase their level of involvement in physical activity, to assess their difficulty in the use of this tool, and to obtain feedback on this. The participants came from a broad age spectrum of the adult population and from different walks of life. Their feedback should be taken into account when considering the implementation of the PAR-Q+ in adult populations working and living in similar urban environments around the world. Language has not been a major issue in this survey since most Singaporeans, in spite of their varied ethnic background, were brought up with the English language as their main medium of education and the main business language in working life.

This study was also noteworthy in that it was able to demonstrate that older age and the presence of certain co-morbidities were not necessarily always associated with less time spent on exercise.

Expectedly, our study demonstrated that co-morbidities generally increased with age. Any pre-participation tool should identify those who are at increased risk. At the same time, the need for every person with a co-morbidity to undergo a medical assessment before exercising is a disincentive to exercise, which is not the intention of such questionnaires. The results from Table 1 also suggest that those in the older age groups and with co-morbidities are spending more time on PA. This would be a positive finding: older age and the presence of co-morbidities should not contra-indicate participation in PA. This also suggests that these individuals may have made more effort to gradually become more physically active for a variety of reasons, such as wisdom of age, more time to spend on physical activity, and the need to overcome and control their co-morbidities or others [18,19,20]. The participants’ responses to the seven PARQ+ main and follow-up questions provide us with data that suggest that age and various co-morbidities need not put a damper on a layperson’s level of PA. This should be useful information when conducting public education on PA and the need to employ due care when increasing one’s level of physical exercise.

The questionnaire should identify those who are especially prone to an acute adverse event, such as cardiovascular collapse or an acute injury. In this study, based on the PARQ+ criteria for referral to a medical practitioner for use of the ePARmedex, 33.1% would have required further work-up. Such large numbers would overtax the health resources of any system. Health care resources are finite and need to be judiciously utilized. How much would a society commit to prevention activities [21]? The question arises whether the PAR-Q+ can be more discriminating in identifying truly at-risk persons. For example, based on this study, if only those who were unable to control their symptoms with their current therapies were referred for medical evaluation, the proportion would have decreased to 10.9% of the population surveyed, which would be less likely to tax the healthcare resources anywhere near as much.

When the PARQ was originally introduced in Canada, it excluded about 20% of the adult population [22] and especially 55% of those aged ≥60 years [23]. With multiple revisions of the PAR-Q+ and subsequent referral to a physician for an ePARmed-X+ evaluation, the exclusion rate has further decreased [24]. What is not known, however, is the proportion of the adult population, especially those above the age of 60 years, who are to be referred for the ePARmed-X+. Even a 20% referral rate after a self-administered tool may seem excessive. The high referral rate seen in our study may also be owing to difficulties in understanding and interpretation of the questions in the PAR-Q+, especially by those who were symptomatic. PA organizers and groups that design pre-participation screening programs need to be mindful of the variety of responses that can be generated with a pre-participation questionnaire that may attempt to widen the number of people who go for medical evaluations prior to participation in sports. Due care in better ensuring that such questionnaires are focused on those who are most at risk will likely generate greater public acceptance.

The major contribution that our study makes to the literature on pre-participation questionnaires would be that a clear and common understanding of such documents drawn up by experts in sports physiology and sports medicine will be needed to better ensure that there is a similar understanding of the language of such questionnaires, especially amongst those who may be more prone to adverse events following PA.

The public feedback suggests that participants with co-morbidities (Table 4) generally found slightly more difficulties in understanding the questions than those who answered no to the various PAR-Q+ and follow-up questions. Table 2 will alert readers to the current phraseology of the PARQ+ and this should help one relate to the results in Table 3 and the comments and suggestions in Table 5. A self-administered pre-participation questionnaire should identify those who matter most, viz., those whose morbidities should be picked up and who should have the least problems understanding the questions. Table 3 of this study points out those questions for which inclusion or exclusion may need to be considered. There will also be a need to review the phraseology of the main PAR-Q+ and follow-up questions to address concerns over length, use of medical jargon, and difficulty in understanding. The suggestions and comments provided in Table 5 can be a resource for the authors of the PARQ+ or other similar questionnaires on how their instrument may be revised.

In a warm and humid environment, moderate to severe PA can result in some degree of dehydration and sometimes heat-related illnesses, such as heat exhaustion and heat stroke, especially for individuals who were unwell for a few days or had a lack of sleep or acclimatization prior to participating in such an activity [1,25]. This factor has not been addressed in the PARQ+.

Increased PA brings on numerous health benefits, including better control of blood sugar and cholesterol and other metabolic disorders, less risk of premature disability and death from cardio-cerebrovascular disease, and improvement in physical fitness levels in a wide variety of persons. A high referral rate for medical evaluation may be a disincentive for those with an initial intention of improving their level of physical and medical fitness. Difficulties in understanding the contents of pre-participation questionnaires can be a disincentive to PA. A slight increase in risk for adverse events may occur with increased PA, at least initially, and precautions would need to be considered to lower these risks, especially in persons unaccustomed to regular exercise. Pre-participation screening is an important component of becoming physically more active. It helps to identify individuals who are at-risk of sudden death or injury [26].

### Limitations of the Study

This study did not examine the reliability of the PAR-Q+ in minimizing the likelihood of adverse events during moderate to intense PA in the community. Such an objective would require a prospective long-term effort involving large numbers of community participants using this tool and monitoring of adverse outcomes after PA. This study also did not address the validity of the use of the seven main questions in the PAR-Q+ in pre-identifying persons at risk. The authors accepted the arguments put forth by the team that drew up the seven main and other follow-up questions, which were evidence based. The public survey that we conducted could not have addressed the issues of reliability and validity.

The study had also combined an apparently disparate group of occupational codes into Occupational Group 3 (unemployed and retired persons, housewives, manual workers, and military persons). This was to address the relatively small numbers in the categories of housewives (37), manual workers (5), and military persons (2) amongst those who participated in the study. The addition of these to Group 3 did not materially affect the analyses of data as regards the duration of physical activity by this group or their feedback to the questions in the PARQ+.

## *5.* Conclusions

Our study suggests that the PAR-Q+ probably over-identifies those who require further medical evaluation to the extent of about one-third of the adult population. The logistical implications of this can be mind-boggling. For the participants, this may be a disincentive to get physically active. The feedback from the participants suggests that the public has difficulty with this tool owing to the perceived wordiness of the document and the frequent use of medical jargon that makes the PARQ+ challenging to understand and use. The suggestions from the potential PA participants provide opportunities to review and revise areas where improvements may be made.

## Figures and Tables

**Table 3:** Feedback on PARQ+ questions.

Main PARQ+ Question and Follow-Up Questions		Ease of Understanding Question (Mean Likert Score ± SD)—Non-Satisficing Participants		*p* Value	Whether No Difficulty, Slight Difficulty, Moderate Difficulty, Significant Difficult
		No Co-Morbidities	With Co-Morbidities		
1.	Has your doctor ever said that you have a heart condition or high blood pressure?	4.74 ± 0.65	4.72 ± 0.63	0.489	No Difficulty
	Do you have a heart of cardiovascular condition? This includes coronary artery disease, heart failure, diagnosed abnormality of heart rhythm	4.34 ± 1.06	4.58 ± 0.93	0.188	Slight difficulty
	i. Do you have difficulty controlling your condition with medication and other physician-prescribed therapies?	4.08 ± 1.16	3.27 ± 1.56	0.235	Significant difficulty
	ii. Do you have an irregular heart rhythm that requires medical management? (e.g., atrial fibrillation, premature ventricular contraction)	4.50 ± 1.04	4.60 ± 0.89	0.857	No difficulty
	iii. Do you have chronic heart failure?	4.27 ± 1.20	3.50 ± 2.12	0.587	Significant difficulty
	iv. Do you have diagnosed coronary artery (cardiovascular) disease and have not participated in regular physical activity in the last 2 months?	4.09 ± 1.31	NA	NA	Slight difficulty
	b. Do you have high blood pressure?	4.81 ± 0.51	4.85 ± 0.49	0.768	No difficulty
	i. Do you have difficulty controlling your condition with medications or other physician-prescribed therapies?	4.20 ± 1.18	3.30 ± 1.74	0.049	Significant difficulty
	ii. Do you have a resting blood pressure ≥ 160/90 mm Hg with or without medication?	4.14 ± 1.30	4.20 ± 1.48	0.647	Slight difficulty
2.	Do you feel pain in your chest at rest, during your daily activities of living, OR when you do physical activity?	4.66 ± 0.78	4.40 ± 1.34	0.855	Slight difficulty
	Do you have respiratory disease? This includes chronic obstructive pulmonary disease, asthma, pulmonary high blood pressure.	3.75 ± 0.84	NA	NA	Moderate difficulty
	i. Do you have difficulty controlling your condition with medication or other physician-prescribed therapies?	NA	NA	NA	
	ii. Has your doctor ever said your blood oxygen level is low at rest or during exercise and/or that you require supplemental therapy?	NA	NA	NA	
	iii. If asthmatic, do you currently have symptoms of chest tightness, wheezing, labored breathing, consistent cough (more than 2 days/week) or have you used your rescue medication more than twice in the last week?	NA	NA	NA	
	iv. Has your doctor ever said that you have high blood pressure in the blood vessels of your lungs?	NA	NA	NA	
3.	Do you lose balance because of dizziness OR have you lost consciousness in the last 12 months? Please answer NO if your dizziness was associated with over-breathing (including during vigorous exercise)	4.59 ± 0.83	4.45 ± 1.04	0.760	Slight difficulty
	Have you had a stroke? This includes Transient Ischemic Attack or Cerebrovascular event.	4.44 ± 0.88	NA	NA	Slight difficulty
	i. Do you have difficulty controlling your condition with medication or other physician-prescribed therapies?	NA	NA	NA	
	ii. Do you have any impairment in walking or mobility?	NA	NA	NA	
	iii. Have you experienced a stroke or impairment of nerves or muscles in the past 6 months?	NA	NA	NA	
	b. Have you experienced a black-out, fainted, or lost consciousness as a result of a head injury within the last 12 months OR have you had a diagnosed concussion within the last 12 months?	4.50 ± 0.97	NA	NA	Slight difficulty
	c. Do you commonly exhibit low resting blood pressure significant enough to cause dizziness, light headedness, and/or fainting?	4.20 ± 0.92	4.50 ± 0.71	0.758	Slight difficulty
	d. Do you have a medical condition such as epilepsy or a neurological condition?	4.80 ± 0.42	NA	NA	No difficulty
4.	Have you been diagnosed with another chronic medical condition (other than heart disease or high blood pressure)?	4.68 ± 0.67	4.55 ± 0.84	0.313	No difficulty
	Do you have kidney problems?	4.80 ± 0.46	NA	NA	No difficulty
	b. Do you have a medical condition that has not been listed above?	4.78 ± 0.67	4.42 ± 1.09	0.711	Slight difficulty
	c. Do you currently live with two or more medical conditions?	4.56 ± 0.96	4.67 ± 0.71	0.942	No difficulty
5.	Are you currently taking prescribed medications for a chronic medical condition? Please list conditions and medications	4.76 ± 0.61	4.76 ± 0.57	0.910	No difficulty
	Do you have any metabolic conditions? This includes Type 1 Diabetes, Type 2 Diabetes, Pre-Diabetes.	4.66 ± 0.68	4.55 ± 0.82	0.944	No difficulty
	i. Do you often have difficulty in controlling your blood sugar levels with foods, medications, or other physician-prescribed therapies?	4.00 ± 1.41	4.00 ± 1.73	1.000	Moderate difficulty
	ii. Do you often, suffer from signs and symptoms of low blood sugar (hypoglycemia) following exercise and/or during activities of daily living? Signs of hypoglycemia may include shakiness, nervousness, unusual irritability, abnormal sweating, dizziness or light-headedness, mental confusion, difficulty speaking, weakness and sleepiness.	4.20 ± 1.32	NA	NA	Slight difficulty
	iii. Do you have signs or symptoms of diabetes complications such as heart or vascular disease and/or complications affecting your eyes, kidneys, OR the sensation in your toes and feet?	4.11 ± 1.36	NA	NA	Slight difficulty
	b. Do you have other metabolic conditions (such as current pregnancy-related diabetes, chronic kidney disease, or liver problems)?	4.48 ± 0.95	NA	NA	Slight difficulty
	c. Are you planning to engage in what for you is unusually high (or vigorous) intensity exercise in the near future?	4.69 ± 0.65	4.71 ± 0.53	0.800	No difficulty
	d. Do you currently have Cancer of any kind?	4.84 ± 0.49	NA	NA	No difficulty
	i. Does your cancer diagnosis include any of the following types: lung/bronchogenic, multiple myeloma (cancer of the plasma cells), head, and/or neck?	NA	NA	NA	
	ii. Are you currently receiving cancer therapy (such as chemotherapy or radiotherapy)?	NA	NA	NA	
	e. Do you have a medical condition that has not been listed above?	4.75 ± 0.64	4.76 ± 0.55	0.876	No difficulty
	f. Do you currently live with two or more medical conditions?	4.65 ± 0.78	4.75 ± 0.65	0.569	No difficulty
6.	Do you currently have (or have within the past 12 months) a bone, joint, or soft tissue (muscle, ligament, or tendon) problem that could be made worse by becoming more physically active?	4.44 ± 0.96	4.41 ± 1.05	0.803	Slight difficulty
	Do you have Arthritis, Osteoporosis, or Back Problems?	4.91 ± 0.38	4.64 ± 0.70	0.052	No difficulty
	i. Do you have difficulty controlling your condition with medication or other physician-prescribed therapies?	4.41 ± 1.10	NA	NA	Slight difficulty
	ii. Do you have joint problems causing pain, a recent fracture or fracture caused by osteoporosis or cancer, displaced vertebra (e.g., spondylolisthesis), and/or spondylolysis/pars defect (a crack in the bony ring on the back of the spinal column)	4.19 ± 1.28	4.13 ± 1.12	0.908	Slight difficulty
	iii. Have you had steroid injections or taken steroid tablets regularly for more than 3 months?	4.74 ± 0.69	4.65 ± 0.78	0.536	No difficulty
	b. Do you have a spinal cord injury? (This includes Tetraplegia and Paraplegia)	4.65 ± 0.94	3.50 ± 2.12	0.000	Significant difficulty
	i. Do you have difficulty controlling your condition with medication or other physician-prescribed therapies?	4.00 ± 1.41	NA	NA	Moderate difficulty
	ii. Has your physician indicated that you exhibit sudden bouts of high blood pressure (known as Autonomic Dysreflexia)?	4.50 ± 0.71	NA	NA	Slight difficulty
7.	Has your doctor ever said that you should only do medically supervised physical activity?	4.78 ± 0.57	NA	NA	No difficulty
	Do you have any Mental Health Problems or Learning Difficulties? This includes Alzheimer’s, Dementia, Depression, Anxiety Disorder, Eating Disorder, Psychotic Disorder, Intellectual Disability, Down Syndrome.	4.61 ± 0.75	NA	NA	No difficulty
	i. Do you have difficulty controlling your condition with medication or other physician-prescribed therapies?	4.65 ± 0.76	NA	NA	No difficulty
	ii. Do you have Down Syndrome AND back problems affecting nerves or muscles?	4.64 ± 0.78	NA	NA	No difficulty

**Table 4:** Frequency of comments and queries by participants based on yes/no responses to questions.

PAR-Q+ Question and Follow-Up Questions	Total Numbers of Queries and Comments	Number (%) of Queries by Participants Who Answered “Yes”	Number (%) of Queries by Participants Who Answered “No”	*p* Value
1	246	70/213 (32.9%)	176/806 (21.8%)	0.001
2	152	5/23 (21.7%)	147/996 (14.8%)	0.432
3	165	14/33 (42.4%)	151/986 (15.3%)	0.000
4	112	23/129 (17.8%)	89/890 (10.0%)	0.008
5	136	33/202 (16.3%)	103/817 (12.6%)	0.167
6	134	41/172 (23.8%)	93/847 (11.0%)	0.000
7	89	0/10 (0.0%)	89/1009(8.8%)	0.326

**Table 5:** Comments, queries, and suggestions by participants on each of the PARQ+ and follow-up questions.

PAR-Q+ Question and Follow-Up Questions	Common Comments (Numbers of Similar Comments if More Than 10)
1	Questions were very wordy with lots of technical terms which are difficult to understand (166) Not sure what is meant by difficulty controlling your condition with medication or other physician-prescribed therapies (73) Should not have two elements in one question, e.g., have diagnosed coronary artery disease, and not participated in regular physical activity in last 2 months (41) Not sure about BP reading of 160/90 mm Hg as most of us do not regularly check our BP. (37) What is a qualified exercise professional? (26)
2	Questions very wordy with lots of technical terms that were difficult to understand (86) Question such as “If asthmatic, do you have chest tightness, wheezing, laboured breathing, consistent cough, or have used rescue medication at least twice over the last 2 months” is confusing, too long and can be broken up into a few sentences with simpler language. (67) Is childhood asthma considered as having asthma? (32) Chest pain can be due to heart or lung problems. Should this be asked as part of Question 1? (21) What is “high blood pressure in the lungs”? (18) Replace “rescue medication” with “inhalers” which is more easily understood. (15) At what reading is oxygen low at rest? (13)
3	Long sentences with multiple issues need to be broken up into smaller statements, e.g., “Do you lose balance because of dizziness?” and “Have you lost consciousness in the last 12 months?” should be two different questions. (96) What is Transient Ischemic Attack? Need to explain in lay terms. (71) What is a cerebrovascular event? Please explain in lay terms (46) How low should blood pressure (BP) be to be reported as low BP? What are low BP symptoms? (33) What does impairment in nerves or muscles mean? (27) The question “Do you have a medical condition such as epilepsy or a neurological condition?” is confusing and should be replaced with a simpler question such as “Do you have a neurological condition such as epilepsy?” (17)
4	Please list examples of chronic medical conditions that we should declare. (48) Questions generally long. Should be shortened. Too many medical terms. Use layman terms. (45) What are chronic medical problems? Please define. Would diabetes, high cholesterol, gastritis, asthma, food allergy, eczema, kidney stones, urinary infection, thyroid problem, scoliosis, hepatitis B carrier all qualify to be chronic medical problems? (37) Why only exclude heart disease and high blood pressure? Why not exclude stroke, dizziness and other conditions covered in previous questions? (17) How will stating my medical problems help me decide whether to go and see a medical practitioner or qualified exercise professional before I change my level of physical activity? (14)
5	Again asking about medical conditions—seems a repetition of previous questions (62) Questions too long-winded and too many medical terms. The longer the question, the more difficult it is to understand. Keep the questions short. (57) To list medications is not easy. Medication names are difficult to remember. Can be omitted? (41) Omit questions about sub-type of cancer. Is this important? (37) Instead of listing cancer types, just state cancer above the waist (20) Do not understand the term multiple myeloma (17) Low blood sugar questions are too long (15) Need to define what is unusually high or vigorous intensity exercise (15) Not too sure what metabolic conditions mean. Is it just diabetes? How about thyroid and problems with other glands? (13) Be specific about sensation in the toes and foot—is it numbness, stiffness, pain or itchiness? (11)
6	Too much use of medical jargon makes this and follow-up questions challenging, unfriendly. 4 out of 8 questions in this section have jargon. Don’t understand bulk of medical terms. (73) When the word “recent” is mentioned, what is the time-line? (38) Long sentences can be shortened and simplified, e.g., Q6 can be read as “In the last 12 months have you had bone, joint or muscle problems that can become worse with physical activity?”. (35) What does “difficulty in controlling your condition” mean? Is it no more pain or less pain? (31) Many older folks have knee pains. Is the PAR-Q+ intended to exclude such persons from physical activity? (26) Does spinal cord injury equal back pain? (14) There are too many follow-up questions. Reduce the number. (12)
7	Not sure how a person with learning difficulties, Alzheimer’s, Down’s, Dementia, Depression, Anxiety Disorder, etc., reliably complete the PARQ+, unless with help of a supervisor. (39) Some questions are too long and with multiple medical jargon. Need simplification. (32) What does medically supervised physical activity mean? Does a doctor need to be present each time for the activity? (22) Do mental health problems also refer to those with ADHD, Autism, Asperger’s, Bipolar disorder. Social anxiety, OCD, etc.? (13)
Additional suggestions	Was participant ill or received vaccination during the 2 weeks before planned exercise? (38) Has any member of the family suffered sudden cardiac death or collapsed while exercising? (29) Any recent injury/surgery within last 3 months that would affect planned physical activity? (23) Did participant get a good night sleep before the planned physical activity? (18) Do you practice self-check, e.g., any dizziness, chest discomfort or unusual shortness of breath, before any moderate or intensive physical activity? (12) To include advisory in the pre-participation questionnaire to include the following: (28) Advice for menopausal or aging women who wish to start exercising Whether have seen a doctor in the last 12 months for a medical check-up? To ensure sufficient hydration before exercising Advice on warming up prior to exercise Rate your willingness to start exercising To provide recommendations on levels of exercise (by minutes per week) What to do if not feeling well during exercise

## Data Availability

The raw data which forms the basis for the Tables in this manuscript is available at https://www.dropbox.com/scl/fo/rx672w1j9w34sk85s2wr9/ADDM-_neBU7-MsbvLpnXemU?rlkey=eoahg15r9cxj2tccul4srbr0j&dl=0 (accessed on 29 April 2024).

## References

[1] Maron B.J., Doerer J.J., Haas T.S., Tierney D.M., Mueller F.O. (2009). Sudden deaths in young competitive athletes: Analysis of 1866 deaths in the United States, 1980–2006. Circulation.

[2] Papadakis M., Sharma S., Cox S., Sheppard M.N., Panoulas V.F., Behr E.R. (2009). The magnitude of sudden cardiac death in the young: A death certificate-based review in England and Wales. Europace.

[3] Thomas S., Reading J., Shephard R.J. (1992). Revision of the Physical Activity Readiness Questionnaire (PAR-Q). Can. J. Sport Sci..

[4] PAR=Q+ The Physical Activity Readiness Questionnaire for Everyone. PAR-Q+ Collaboration. https://eparmedx.com/?page_id=75.

[5] Sports Safety Committee (2007). Overview and Recommendations for Sports Safety in Singapore-A Report by the Sports Safety Committee. https://www.sportsingapore.gov.sg/files/Sport%20Education/Sport%20Safety/Sports_Safety_Committee_26SEPO7.pdf.

[6] Canadian Society for Exercise Physiology (2017). Get Active Questionnaire. https://csep.ca/2021/01/20/pre-screening-for-physical-activity/#post/0.

[7] Tremblay M.S., Warburton D.E., Janssen I., Paterson D.H., Latimer A.E., Rhodes R.E., Kho M.E., Hicks A., LeBlanc A.G., Zehr L. (2011). New Canadian physical activity guidelines. Appl. Physiol. Nutr. Metab..

[8] Warburton D.E., Charlesworth S., Ivey A., Nettlefold L., Bredin S.S. (2010). A systematic review of the evidence for Canada’s Physical Activity Guidelines for Adults. Int. J. Behav. Nutr. Phys. Act..

[9] Warburton D.E.R., Katzmarzyk P.T., Rhodes R.E., Shephard R.J. (2007). Evidence-informed physical activity guidelines for Canadian adults. Appl. Physiol. Nutr. Metab..

[10] Maranhao Neto G.A., Luz L.G., Farinatti P.T. (2013). Diagnostic accuracy of pre-exercise screening questionnaire: Emphasis on educational level and cognitive status. Arch. Gerontol. Geriatr..

[11] Warburton D.E.R., Jamnik V.K., Bredin S.S.D., Gledhill N., PAR-Q+ Research Collaboration (2011). The Physical Activity Readiness Questionnaire (PAR-Q+) and electronic Physical Activity Readiness Medical Examination (ePARmed-X+). Health Fitness J. Can..

[12] Humphrey R.A., Lakomy J. (2003). An evaluation of pre-exercise screening questionnaires used within the health and fitness industry in the United Kingdom. Phys. Ther. Sport.

[13] Sport Singapore (2019). Sport Safety Committee Report. https://www.sportsingapore.gov.sg/files/Sport%20Education/Sport%20Safety/2019_Sports_Safety_Committee_Report_30Oct19(4).pdf.

[14] WHO Expert Consultation (2004). Appropriate body-mass index for Asian populations and its implications for policy and intervention strategies. Lancet.

[15] International Labour Organisation Department of Statistics. International Standard Classification of Occupations (ISCO). https://ilostat.ilo.org/resources/concepts-and-definitions/classification-occupation/.

[16] Barge S., Gehlbach H. (2012). Using the Theory of Satisficing to Evaluate the Quality of Survey Data. Res. High. Educ..

[17] Vriesema C.C., Gehlbach H. (2021). Assessing survey satisficing: The impact of unmotivated questionnaire responding on data quality. Educ. Res..

[18] Goodman J.M., Burr J.F., Banks L., Thomas S.G. (2016). The Acute Risks of Exercise in Apparently Healthy Adults and Relevance for Prevention of Cardiovascular Events. Can. J. Cardiol..

[19] Liguori G., Feito Y., Foutaine C., Roy B. (2021). ACSM’s Guidelines for Exercise Testing and Prescription.

[20] Buchbinder R., Underwood M., Hartvigsen J., Maher C.G. (2020). The Lancet Series call to action to reduce low value care for low back pain: An update. Pain.

[21] Mont L., Pelliccia A., Sharma S., Biffi A., Borjesson M., Terradellas J.B., Carré F., Guasch E., Heidbuchel H., Gerche A. (2017). Pre-participation cardiovascular evaluation for athletic participants to prevent sudden death: Position paper from the EHRA and the EACPR, branches of the ESC. Endorsed by APHRS, HRS, and SOLAECE. Europace.

[22] Shephard R.J., Cox M.H., Simper K. (1981). An analysis of “Par-Q” responses in an office population. Can. J. Public Health.

[23] Shephard R.J., Mahoney A., Flowers J.F., Benidge M.E. (1984). On the preliminary screening of elderly exercise volunteers. Can. J. Aging.

[24] Bredin S.S., Gledhill N., Jamnik V.K., Warburton D.E. (2013). PAR-Q+ and ePARmed-X+: New risk stratification and physical activity clearance strategy for physicians and patients alike. Can. Fam. Physician.

[25] Nichols A.W. (2014). Heat-related illness in sports and exercise. Curr. Rev. Musculoskelet. Med..

[26] Corrado D., Schmied C., Basso C., Borjesson M., Schiavon M., Pelliccia A., Vanhees L., Thiene G. (2011). Risk of sports: Do we need a pre-participation screening for competitive and leisure athletes?. Eur. Heart J..

